# High-quality metagenome assembly from long accurate reads with metaMDBG

**DOI:** 10.1038/s41587-023-01983-6

**Published:** 2024-01-02

**Authors:** Gaëtan Benoit, Sébastien Raguideau, Robert James, Adam M. Phillippy, Rayan Chikhi, Christopher Quince

**Affiliations:** 1 Organisms and Ecosystems, Earlham Institute, Norwich, UK; 2 Gut Microbes and Health, Quadram Institute, Norwich, UK; 3 Genome Informatics Section, National Human Genome Research Institute, Bethesda, MD USA; 4 Sequence Bioinformatics, Department of Computational Biology, Institut Pasteur, Paris, France; 5 School of Biological Sciences, University of East Anglia, Norwich, UK; 6 Warwick Medical School, University of Warwick, Coventry, UK

**Keywords:** Metagenomics, Genome assembly algorithms

## Abstract

We introduce metaMDBG, a metagenomics assembler for PacBio HiFi reads. MetaMDBG combines a de Bruijn graph assembly in a minimizer space with an iterative assembly over sequences of minimizers to address variations in genome coverage depth and an abundance-based filtering strategy to simplify strain complexity. For complex communities, we obtained up to twice as many high-quality circularized prokaryotic metagenome-assembled genomes as existing methods and had better recovery of viruses and plasmids.

## Abstract (web summary)

An assembler considering variable coverage depths increases the number of near-complete metagenome-assembled genomes.

## Main

Shotgun metagenomics, the sequencing of DNA from a mixed sample of genomes in a community^1–3^, provides a high-throughput means to survey microbial population genomic diversity. A critical first step in metagenomics analyses is the assembly of shotgun reads into longer contiguous sequences or contigs. Genome assemblies that are derived from short reads can be highly fragmented into potentially millions of contigs per sample, particularly if they are from diverse communities. The difficulty in assembling metagenomes is a consequence of intra- and inter-genome sequence repeats, low coverage of some species and strain diversity^4,5^. Many complete genomes are, nevertheless, recovered by clustering (binning) short contigs using features such as sequence composition or differential coverage across multiple samples^6^, creating metagenome-assembled genomes (MAGs). Although MAGs have resulted in thousands of bacterial genomes being added to reference databases, MAGs from short-read metagenomes are often fragmented, contaminated and missing key regions such as the 16S rRNA gene operon.

Third-generation long-read sequencing technologies have greatly improved the quality of metagenome assemblies and MAGs. The first applications, using reads generated by the Oxford Nanopore Technologies (ONT) platform (which, at that point, had a relatively high error rate) typically only resolved a small fraction of the community as complete circularized contigs^7^. More recent ONT studies have generated hundreds of MAGs but only a relatively small number of closed circularized genomes^8,9^. An alternative long-read technology, HiFi PacBio, combines long reads with very high accuracies (≈99.9%). This has enabled hundreds of MAGs to be retrieved from metagenomes with a substantially larger fraction as circularized contigs^10^. An important caveat is that platform comparisons cannot easily be made across studies owing to variations in sequencing depth and community complexity^8^.

Existing algorithms for metagenomics assembly of HiFi PacBio reads are effective but have limitations. Firstly, both low-abundance and high-abundance organisms with strain diversity may not be assembled^11^ and, hence, the majority of the community by abundance will not be resolved as high-quality MAGs^10^. Secondly, even typical metagenomes require long processing times (days) and high-end computing infrastructure (>500 GB to 1 TB of memory), and therefore scaling to larger data sets from more complex communities is prohibitive. Thirdly, they do not allow the easy incorporation of contextual data such as depth of coverage, which is a critical component in metagenome reconstruction.

There are two generally accepted paradigms for sequence assembly: string graph methods that operate with individual reads, which consider pair-wise overlaps and construct graphs to represent them^12^, and de Bruijn graph (DBG) assemblers, in which reads are first decomposed into short, fixed-length sequences (*k*-mers)^13^. The former requires all-versus-all read comparisons, which scales poorly with read numbers and hence is too inefficient for short-read metagenomics. It has been applied to long reads, specifically HiFi PacBio metagenomics, in hifiasm-meta ^14^, using minimizers to efficiently find read overlaps. String graphs, although they are effective, will always scale poorly with large read numbers, and the complex graphs that are generated make coverage estimation difficult because of ambiguous read mapping.

The decomposition to *k*-mers in DBG assemblers enables them to reduce the volume of data that is being processed and efficiently detect overlaps; consequently, they are now the default for short reads. However, there are two challenges in applying DBGs to long reads. Firstly, they effectively assume exact overlaps. Secondly, for long reads, the required overlap (and therefore *k*-mer size) becomes large and the number of unique *k*-mers required (and therefore memory required) becomes prohibitive. A hybrid approach has been developed (Flye^15^) that uses a form of sparse DBG^16^ to assemble noisy disjointigs, which are then used to create a repeat graph that is further resolved through read mapping. This works for both Nanopore and HiFi PacBio sequences and has been adapted to metagenomics; however, it also does not scale particularly well and produces inferior results compared to hifiasm-meta on HiFi data^10,17^.

A fundamentally different approach to the problem of adapting DBGs to long reads was introduced with rust-mdbg^18^. This implementation uses a minimizer-space DBG (MDBG) in which the *k*-mers are replaced by sequences of universal minimizers, which are *k*-mers that map to an integer below a fixed threshold and are a means of reproducibly subsampling *k*-mers. The result is a graph that is more sparse and lightweight: for example, just 12 million nodes are required to assemble a complete human genome. It can also deal better with noise than long-nucleotide *k*-mer DBGs because exact matches are only required for the small selected minimizers. The rust-mdbg algorithm is, however, not designed for metagenomics. In particular, it cannot cope well with variable genome coverage depths.

**Fig. 1:**
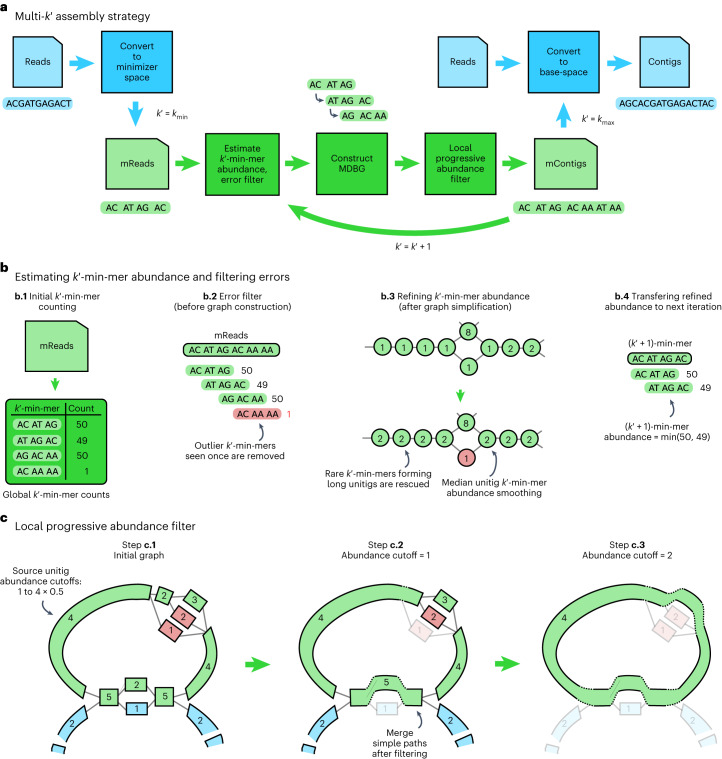
Overview of the algorithmic steps of metaMDBG. **a**, Overview of the multi-${k}^{{\prime} }$ assembly strategy. Processes in blue are performed at the level of nucleotide sequences and those in green are performed only at the level of minimizers. **b**, Components for estimating and refining ${k}^{{\prime} }$-min-mer abundance as ${k}^{{\prime} }$ is increased and for filtering errors before graph construction. **c**, Illustration of the 'local progressive abundance filter' algorithm that simplifies complex graph regions generated by errors, inter-genomic repeats and strain variability. Each node represents a unitig (unitigs in green and blue belong to two distinct species and unitigs in red represent errors). The long unitig (with abundance = 4) is chosen as the seed (step **c.1**). Its abundance is used as a reference to apply a 'local progressive abundance filter' from 1× to 0.5× its abundance (steps **c.2** and **c.3**). At each step, unitigs with abundance equal to the cutoff value are removed and the graph is re-compacted to simplify fragmented unitigs. Note that fragmented green unitigs with abundance = 2 would have been removed without the intermediate step **c.2**.

We introduce metaMDBG (see Methods and Fig. 1a), which takes the principle of minimizer-space assembly and engineers it specifically for metagenomics from high-fidelity long reads. Each read is first converted into a minimizer-space read (mRead), which is an ordered list of minimizers. Each iteration of the assembler then comprises the construction of a DBG using lists of minimizers of fixed length (${k}^{{\prime} }$), denoted ${k}^{{\prime} }$-min-mers. Following filtering of low-frequency ${k}^{{\prime} }$-min-mers, the graph is constructed and simplification is performed using standard methods (for example, tip clipping and bubble popping).

We exploit the ease with which abundance estimates can be obtained for ${k}^{{\prime} }$-min-mers to integrate abundance information directly inside the assembly algorithm. This ‘local progressive abundance filter’ removes complex errors, inter-genomic repeats and strain variability (Fig. 1c). It starts by identifying long seed unitigs and then increments an abundance threshold from one up to 50% of the seed coverage depth. At each step, unitigs with coverage equal to or lower than the threshold are removed, the graph is re-compacted and unitig coverage estimates are refined (Fig. 1b).

These algorithmic advances are integrated within a highly efficient multi-*k* approach that is entirely in minimizer space, and they address the variable coverage depths found in metagenomes. The minimizer-space contigs (mContigs) from the last iteration are added to the set of input mReads in the next iteration and these steps are repeated after incrementing ${k}^{{\prime} }$. At the end of the multi-*k* process, reads are mapped to the final mContigs to determine their base-space sequence. This is followed by a low-memory re-implementation of the racon^19^ contig-polishing strategy and purging of strain duplicates.

### Benchmarking setup

We compared metaMDBG with two other state-of-the-art assemblers for HiFi metagenomics data, metaFlye (v.2.9-b1768) and hifiasm-meta (v.0.2-r058), on two mock communities and three real metagenomes (Supplementary Table S1). The commands that were used are provided in Supplementary Table S2 and all assembly results are summarized in Supplementary Table S3. A comparison to rust-mdbg is also given, although only on a subset of the data sets as explained below. The two mock communities, ATCC^20^ and Zymo, contain 20 and 21 species, respectively, for which abundances and reference genomes are known (see Supplementary Table S4). The first real metagenome, ‘human gut’, is a PacBio HiFi-generated data set composed of four human fecal samples from omnivore and vegan donors^14^. The second metagenome, ‘AD-HiFi’, is a time series of three samples that were extracted from anaerobic digester sludge and generated for this study. For these two projects, where multiple samples were available, we present results from the co-assemblies of all samples together. The third data set, ‘sheep rumen’, is a single deeply sequenced sample from the sheep rumen^10^.

### Improved recovery of complete circularized genomes

We first evaluated the assemblers on two mock communities, Zymo-HiFi and ATCC, by aligning contigs to references and computing average nucleotide identity (see Methods). The results are summarized in Supplementary Table S4. Rust-mdbg was not competitive with the other assemblers. This is not surprising, as rust-mdbg performs no polishing and is designed specifically for rapid and draft-level genome assembly; therefore, we will exclude it from the further comparisons below. MetaMDBG performed similarly to hifiasm-meta and metaFlye, both in terms of the number of species obtained as circularized contigs and the average nucleotide identity to reference sequences (>99.99% in most cases). The Zymo-HiFi mock community contains 21 genomes, but five have very low coverage and five are strains of *E. coli*. In this case, metaMDBG and hifiasm-meta both obtained ten circularized genomes and metaFlye obtained nine; however, metaMDBG additionally generated two almost complete (>99.8%) genomes as linear contigs. No assembler could correctly resolve all of the *E. coli* strain diversity; however, metaMDBG and hifiasm-meta each succeeded in circularizing one strain, and the latter had all the other strains present as fragmented contigs, whereas metaFlye produced only fragmented genomes. The mock ATCC community contains 20 species, but only 15 were obtained by any of the assemblers, probably because the others lacked sufficient coverage depth. Of these species, each assembler obtained 12 as circularized contigs, although not the same 12, and each assembler assembled one species uniquely.

**Fig. 2:**
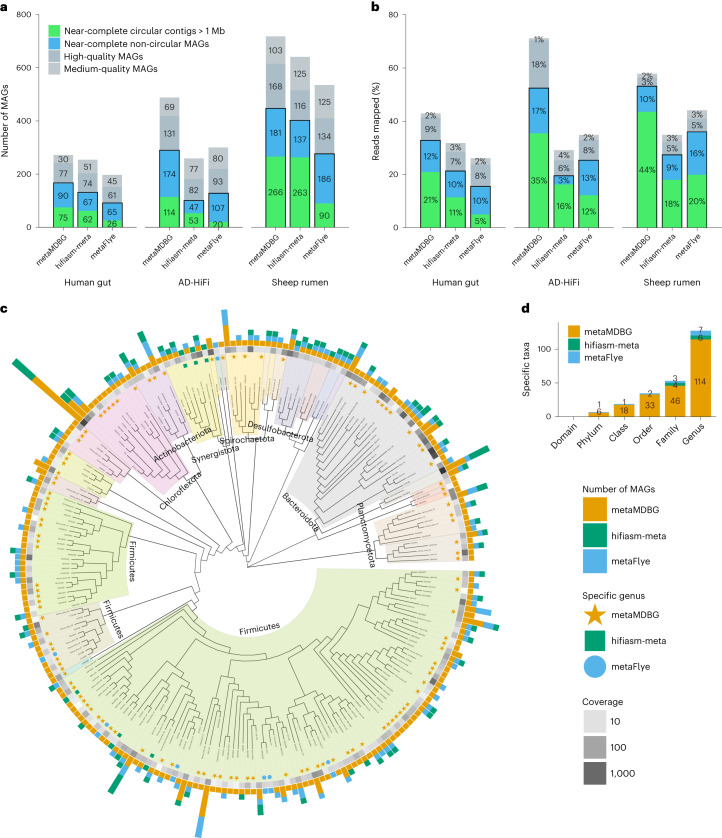
Assembly results on three HiFi PacBio metagenomic projects. **a**, CheckM evaluation. A MAG is considered 'near-complete' if its completeness is ≥90% and contamination is ≤5%; 'high quality' if its completeness is ≥70% and contamination is ≤10%; and 'medium quality' if its completeness is ≥50% and contamination is ≤10%. **b**, The percentage of mapped HiFi reads on MAGs. **c**, Phylogenetic tree of genera recovered from the AD-HiFi data set for all assemblers combined. For the near-complete bacterial MAGs, we generated a de novo phylogenetic tree based on GTDB-Tk marker genes, displayed at the genus level. The outer bar charts give the number of MAGs found in each genus. The colored symbols then denote genera recovered by only one of the assemblers. The grayscale heat map illustrates the aggregate abundance of dereplicated MAGs in a genus. **d**, Number of taxa at different levels that are unique to each assembler.

For the real communities, we used CheckM (v.1.1.3) to obtain the level of genome completeness and contamination of each contig and determine whether they are MAGs. These were then grouped as ‘near-complete’ (completeness ≥ 90%, contamination ≤ 5%), ‘high quality’ (completeness ≥ 70%, contamination ≤ 10%) and ‘medium quality’ (completeness ≥ 50%, contamination ≤ 10%). For all three real communities, we observe a significant improvement in the number of circularized near-complete MAGs (cMAGs) longer than 1 Mb generated by metaMDBG compared to the state-of-the-art algorithms (Fig. 2a). MetaMDBG assembled 75 cMAGs from the human gut microbiome data set (13 more than hifiasm-meta), 114 from the AD-HiFi data set (61 more than hifiasm-meta) and 266 from the sheep rumen data set (three more than hifiasm-meta). MetaFlye produced significantly fewer cMAGs than the other two assemblers. As a further validation of the quality of the cMAGs, we predicted the presence of rRNA and tRNA genes (see Methods). This step confirmed that HiFi cMAGs usually do contain the expected complement of RNA genes (96.0%, 96.6% and 98.5% of metaMDBG, hifiasm-meta and metaFlye MAGs, respectively) and that all three assemblers generate cMAGs of similarly high quality.

**Extended Data Fig. 1:**
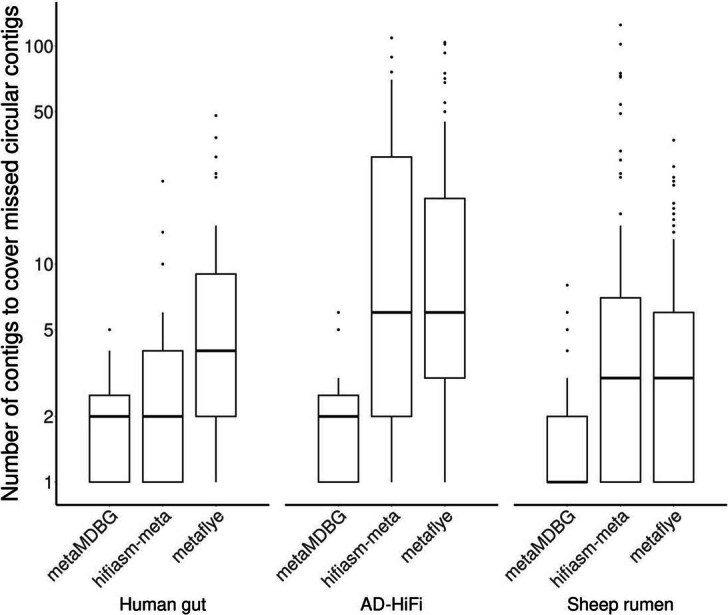
Number of contigs required to cover a near-complete circular MAG reconstructed successfully by an alternative assembler. In order to estimate the degree of fragmentation of assemblers, we aligned the contigs of one assembler against the near-complete circular contigs (cMAGs) recovered by the other assemblers. The fragmentation is then represented as the number of contigs required to cover these cMAGs (see section ‘Assessment of completeness and fragmentation of assemblies using reference sequences’ for details). The boxplot elements are the median (horizontal bar), 25th and 75th percentiles (box limits Q1 and Q3), Q1-1.5*IQR and Q3+1.5*IQR (whiskers, IQR=Q3-Q1) and outliers. Summary statistics (n, min, median, mean, max): Human gut- metaMDBG (19, 1, 2, 2.1, 5); hifiasm-meta (32, 1, 2, 4, 24); metaFlye (68, 1, 4, 7.5, 48) : AD-HiFi- metaMDBG (11, 1, 2, 2.3, 6); hifiasm-meta (72, 1, 6, 19.8, 109); metaFlye (105, 1, 6, 15.1, 104) : Sheep rumen- metaMDBG (15, 1, 1, 1.8, 8); hifiasm-meta (18, 1, 3, 10.7, 125); metaFlye (183, 1, 3, 5, 37). The data to generate this boxplot have been extracted from Supplementary Table S5.

**Extended Data Fig. 2:**
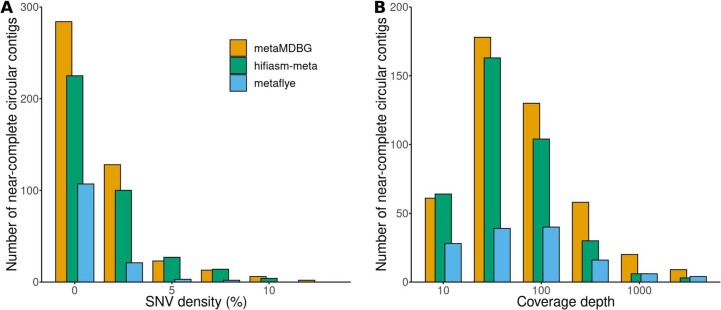
Histograms of SNV density and coverage depths for near-complete circular contigs. SNV densities (A) and coverage depths (B) are shown for all the near-complete circular contigs (see definition in text) aggregated across the three HiFi PacBio datasets (Human gut, AD-HiFi, Sheep Rumen) for each assembler (metaMDBG, hifiasm-meta, metaFlye).

To investigate differences between the cMAGs generated by the assemblers, we aligned the assemblies against each other with wfmash (Supplementary Tables S5 and S6) and computed their coverage depth and single-nucleotide variant (SNV) density (see Methods). In the sheep rumen data set, metaMDBG and hifiasm-meta combined found a total of 356 distinct near-complete circular contigs. Among them, 176 were found by both assemblers (49%), with 90 specific to metaMDBG and 87 specific to hifiasm-meta. The majority (91%) of these specific cMAGs are still present in the other assemblies but as one or more linear contigs. The cMAGs missed by metaMDBG were less fragmented, with a median of one contig (mean, 1.3) necessary to cover a cMAG reconstructed by another assembler, compared to a median of three contigs for both hifiasm-meta (mean, 10.7) and metaFlye (mean, 5) (see Extended Data Fig. 1). In the human gut microbiome and AD-HiFi data sets, we observed similar results in terms of reduced linear contig fragmentation for metaMDBG. MetaMDBG and hifiasm-meta were able to generate cMAGs across a range of SNV densities (Extended Data Fig. 2A), but we found a highly significant negative relationship between SNV density and the probability that metaFlye would assemble a cMAG (logistic regression coefficient, –1.35, *P* = 2.18 × 10^–11^, *n* = 575). For intermediate coverage depths, metaMDBG and hifiasm-meta had similar success at resolving cMAGs; however, at higher coverages, more than twice as many cMAGs were obtained by metaMDBG (Extended Data Fig. 2B).

### Improved reconstruction of phages and plasmids

We used viralVerify^21^ to identify the circular components that were potentially plasmid or phage genomes (Supplementary Table S7). MetaMDBG identified substantially more circularized plasmids and phages than hifiasm-meta, which was second best for all three metagenomes (sheep rumen, 70% more circularized plasmids and 25% more phages; human gut coassembly, 42% more plasmids and 55% more phages; AD-HiFi coassembly, more than twice as many plasmids and 78% more phages). For the circularized phages, we used CheckV^22^ to determine predicted completeness. We found that 39.4% and 45.2% of the genomes were judged as high quality for metaMDBG and hifiasm-meta, respectively, but we still obtained substantially more (25.8%) high-quality phage genomes with metaMDBG because of the greater number of initial predictions.

### Recovery of a majority of communities as near-complete MAGs

**Extended Data Fig. 3:**
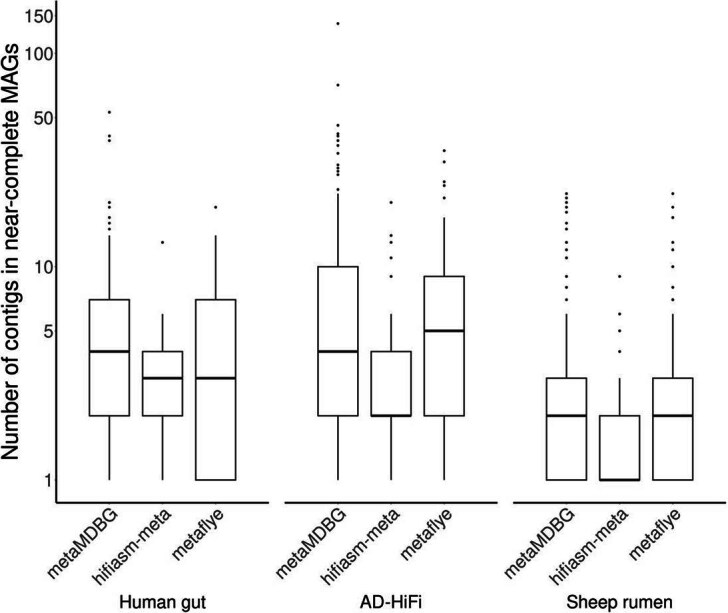
Number of contigs in non-circular near-complete MAGs. The boxplot elements are the median (horizontal bar), 25th and 75th percentiles (box limits Q1 and Q3), Q1-1.5*IQR and Q3+1.5*IQR (whiskers, IQR=Q3-Q1) and outliers. Summary statistics (n, min, median, mean, max): Human gut- metaMDBG (90, 1, 4, 6.8, 53); hifiasm-meta (67, 1, 3, 3.1, 13); metaFlye (65, 1, 3, 4.6, 19) : AD-HiFi- metaMDBG (174, 1, 4, 9.2, 138); hifiasm-meta (47, 1, 2, 3.6, 20); metaFlye (107, 1, 5, 7, 35) : Sheep rumen-metaMDBG (181, 1, 2, 3.3, 22); hifiasm-meta (137, 1, 1, 1.5, 9); metaFlye (186, 1, 2, 2.8, 22). The data to generate this boxplot have been extracted from Supplementary Table S5.

To date, no HiFi PacBio assembler has succeeded in recovering the majority of a complex microbial community by abundance as near-perfect MAGs. To reconstruct non-circular MAGs, we binned contigs from each assembler with MetaBAT2, using sequence composition and coverage after first subtracting all circularized contigs of ≥1 Mb (see Methods). The contigs that are removed before binning will include the cMAGs identified above, as this ensures that bins are constructed only from genome fragments^14^. We then evaluated these bins with CheckM (see Supplementary Table S8 for the list of MAGs). MetaMDBG reconstructed 23 (34%) more near-complete non-circular MAGs than hifiasm-meta in the human gut coassembly, 127 (270%) more in the AD-HiFi time series and 44 (32%) more in the sheep rumen data set (Fig. 2a). MetaFlye produced fewer near-complete circular contigs than the other assemblers but an equivalent or higher number of near-complete or high-quality MAGs compared to hifiasm-meta across all data sets and an equivalent or lower number compared to metaMDBG. The non-circular near-complete MAGs from all assemblers typically contained less than ten contigs (Extended Data Fig. 3).

**Extended Data Fig. 4:**
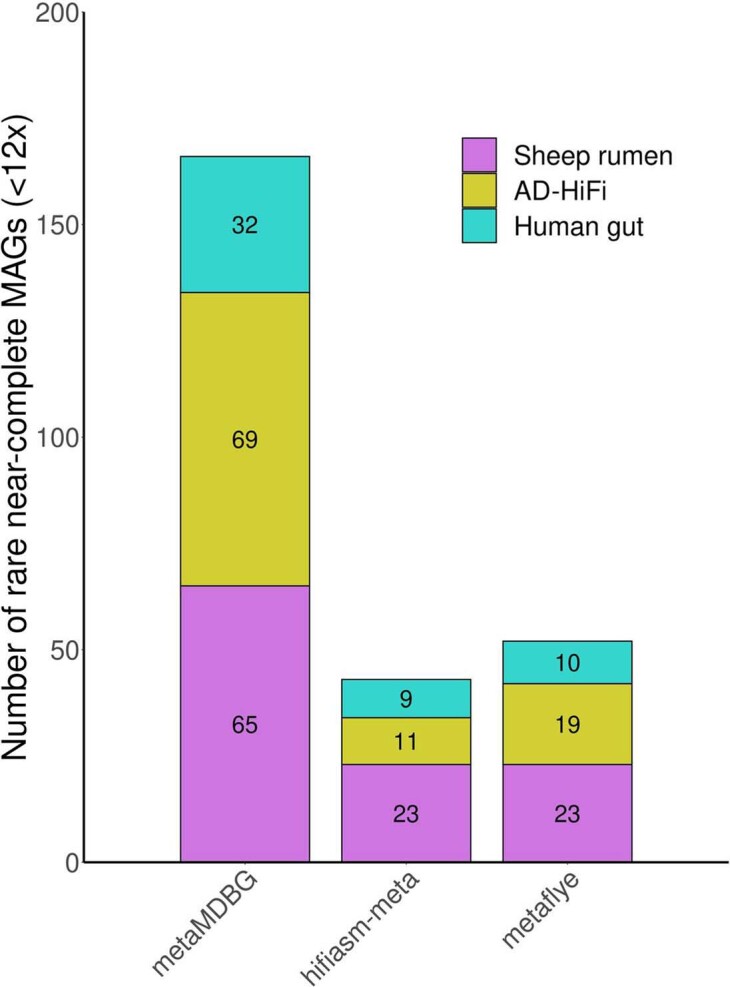
Number of low-coverage non-circular near-complete MAGs recovered by the assemblers. For the three tested PacBio HiFi datasets, we show the number of non-circular near-complete MAGs with low coverage ( < 12x) reconstructed by each assembler.

**Extended Data Fig. 5:**
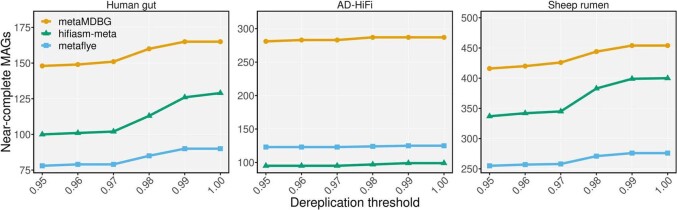
Total number of near-complete MAGs (circular and non-circular) across different dereplication thresholds. We used dRep to cluster MAGs by nucleotide similarity using the parameter -sa from 0.95 to 1. This Figure shows for each assembler on each data set, how the number of dereplicated near-complete MAG clusters, both circular and non-circular, collapses as they are dereplicated at decreasing levels of nucleotide similarity. In the Sheep rumen and Human gut data sets, the number of dereplicated MAG clusters from hifiasm-meta drops significantly below a 97% ANI dereplication threshold, this is not observed for metaMDBG or metaFlye, which indicates that a greater proportion of the hifiasm-meta MAG diversity is at the strain-level. This is not the case for the AD-HiFi data set where no assembler seems to generate a substantial number of strains with more than 97% ANI.

The improvement in the number of near-complete non-circular MAGs produced by metaMDBG is mainly a result of better recovery of low-abundance organisms (Extended Data Fig. 4). This, combined with the higher number of abundant cMAGs, means that for the AD-HiFi and sheep rumen data sets, metaMDBG succeeds in obtaining a collection of near-complete MAGs that can map over 50% of reads (Fig. 2b). This was not the case for the human gut data set, which may be a consequence of the relatively lower depth of sequencing. The assemblers also differ in the nucleotide divergence of the near-complete MAGs that they resolve (Extended Data Fig. 5). In the sheep rumen and human gut data sets, a greater proportion of the hifiasm-meta MAG diversity is at the strain level.

To summarize the microbial diversity from the AD-HiFi coassembly, we constructed a phylogenetic tree at the genus level (see Methods) for all near-complete MAGs from all assemblers (Fig. 2c). The improved MAG recovery by metaMDBG translates into a more representative picture of microbial diversity at all levels of evolutionary divergence. In total, we observed 114 genera that were recovered from the AD-HiFi data sets by metaMDBG but are missing from the near-complete MAG collections of the other programs. When the other assemblers did recover MAGs from the same genus, in all but one case metaMDBG found more MAGs. Finally, we can see large parts of the tree in Fig. 2c that are represented by only metaMDBG MAGs; indeed, six phyla (46 families) were found only by metaMDBG, compared to one phylum (4 families) specific to metaFlye and four families specific to hifiasm-meta (see Fig. 2d and Supplementary Table S9). The phyla that are unique to the metaMDBG near-complete MAGs include recently discovered phyla, with no cultured representatives (for example, OLB16 (ref. ^23^) and *Riflebacteria*^24^).

### Efficient large-scale assembly

MetaMDBG is highly scalable, both in terms of execution time and memory footprint (Supplementary Table S3). MetaMDBG took 36 h to complete the human gut data set, which is 20% faster than the other assemblers. This gain increased substantially with the more complex sheep rumen and AD-HiFi data sets. MetaMDBG took about 3 days to assemble the AD-HiFi data sets compared to 8 days for metaFlye and 39 days for hifiasm-meta. We observed a similar trend with the sheep rumen data set. With regards to memory usage, metaMDBG required only 14 GB to assemble the human gut data set, whereas metaFlye and hifiasm-meta used more than 130 GB. The memory consumption of metaMDBG for the AD-HiFi and sheep rumen samples spiked at only 16 GB and 22 GB, respectively, despite the larger diversity detected in those data sets. The memory usage of MetaFlye (650 GB) and hifiasm-meta (800 GB) was many times this amount.

### Evaluation of metaMDBG for ONT metagenome assembly

MetaMDBG is optimized for HiFi PacBio reads but the accuracy of the more cost-effective ONT reads is continuously improving; therefore, we also evaluated metaMDBG on two recently generated Oxford Nanopore R10.4 data sets^8^ (Supplementary Table S1). The first data set, Zymo-ONT (a simplified version of the Zymo-HiFi mock considered above, comprising seven bacterial species and one fungus) sequenced to high depth with an estimated per-base accuracy of 99.14%. Both metaMDBG and metaFlye assembled all the bacterial species as circularized contigs at high average nucleotide identity (median, 99.99%) and completeness (median, 99.97%). Hifiasm-meta produced only fragmented genomes; however, it is not designed for ONT reads and therefore we will not discuss its results further (see Supplementary Table S10). The second data set, AD-ONT (from an anaerobic digester) had effectively much lower coverage, as only 14 Gbp of reads were generated from a far more complex community at an observed accuracy of 98.11%. Here, on raw reads, metaFlye outperformed metaMDBG in terms of MAGs, obtaining 42 near-complete or high-quality MAGs as opposed to just seven. The results were more comparable after polishing with the Nanopore reads themselves. Using VeChat before assembly^25^, we then obtained 28 near-complete or high-quality MAGs from metaMDBG versus 34 for metaFlye (see Supplementary Table S11). Furthermore, using short reads from the same community to polish the Nanopore reads with Ratatosk^26^, we saw a clear performance benefit with metaMDBG, obtaining 52 near-complete or high-quality MAGs versus 32 for metaFlye. In addition, eight of the near-complete MAGs generated by metaMDBG were circularized compared to just one generated by metaFlye.

### Summary

We have introduced metaMDBG, an assembler for long and accurate metagenomics reads based on the MDBG. Our aim was to develop a scalable assembler for high-fidelity long reads. We succeeded in this goal, as metaMDBG, tested on a range of HiFi PacBio data sets, was 1.5 to 12 times faster than the state of the art and required between one-tenth and one-thirtieth of the memory. Moreover, we achieved this result with substantially better assembly results, particularly in strain-diverse communities such as the AD-HiFi data set, and we succeeded in reconstructing the majority of communities by abundance as near-complete MAGs. We also demonstrated improved results for phages and plasmids. We could not demonstrate an improvement for raw ONT reads; however, if short reads are available to error-correct before assembly, then we can obtain more high-quality MAGs and, in particular, more circularized MAGs. In summary, we have demonstrated the power of MDBGs for the assembly of highly accurate long reads from metagenomes. We believe that further advances in our methodology coupled with larger data sets will greatly contribute to achieving complete genome-scale resolution of even the most complex metagenomes.

## Methods

### Overview of metaMDBG

We present metaMDBG, a method for assembling metagenomes from accurate long reads (for example, PacBio HiFi). MetaMDBG takes as input a set of reads and outputs a FASTA file with contigs. The overall assembly strategy is summarized in Fig. 1a. The universal minimizers, which are *k*-mers that map to an integer below a fixed threshold (see below), are first identified in each read. Each read is thus represented as an ordered list of the selected minimizers, denoted an mRead. Each iteration of the assembler then comprises the construction of a DBG using lists of minimizers of fixed length, ${k}^{{\prime} }$ (denoted ${k}^{{\prime} }$-min-mers), starting with ${k}_{\rm{min}}^{{\prime} }=4$. We count ${k}^{{\prime} }$-min-mers across the whole data set, and those with a frequency below a set threshold are filtered (Fig. 1b). The graph is then constructed and graph simplification is performed. This process includes classical methods for contig generation, such as tip clipping and bubble popping. Following this step, a ‘local progressive abundance filter’ is performed to remove potential inter-genomic repeats, strain variability and complex error patterns (Fig. 1c), beginning by identifying long seed unitigs (long, non-branching paths in the graph). We then increment an abundance threshold starting at one up to 50% of the coverage depth of this seed. At each step, unitigs with coverage equal to or lower than the threshold are removed and the graph is re-compacted. This strategy, coupled with techniques for refining unitig coverage estimation (Fig. 1b), enables the seed unitig to converge conservatively on its longest possible form as complexity is removed from the graph. At this stage, one iteration in our multi-${k}^{{\prime} }$ approach in this minimizer space is complete. The resulting mContigs are added to the set of input mReads in the next iteration, and these steps are repeated after increasing ${k}^{{\prime} }$ by an increment of one. At the end of the multi-${k}^{{\prime} }$ process, when ${k}^{{\prime} }$ equals ${{k}^{{\prime} }}_{\rm{max}}$, reads are mapped to the final mContigs in order to extract their base-space sequence. This is followed by a low-memory re-implementation of the racon^19^ polishing strategy and purging of strain duplicates.

### Preliminaries

We start with a lexicon of some terms and concepts related to MDBGs and genome assembly.

#### Minimizer

In this work, we adopt the concept of a universal minimizer as previously defined^18^. Recall that in the original definition of minimizers^27^, a window is used to compute minimizers. Universal minimizers are pre-determined and do not require a window to be defined. Specifically, let *f* be a function that takes as input a *k*-mer (string of size *k*) and outputs an integer value within the range [0, *H* [, where *H* is typically equal to 2^64^. Given 0 < *d* < 1 and *k* > 0, a universal minimizer is any string *m* of length *k* over the DNA alphabet such that *f*(*m*) < *dH*. The value of *d* represents the density of *k*-mers that will be considered as minimizers over the space of all possible *k*-mers.

#### Minimizer-space read

Before MDBG construction, each read is scanned and its minimizers are identified. Each read is therefore represented as an ordered list of minimizers. We call this minimizer representation of a read the mRead.

#### *k*′-min-mer

A ${k}^{{\prime} }$-min-mer is a list of ${k}^{{\prime} }$ successive minimizers. They are collected by sliding a window of size ${k}^{{\prime} }$ over the mReads.

#### MDBG

The MDBG is constructed from the set of ${k}^{{\prime} }$-min-mers. An MDBG is a directed graph in which the nodes are ${k}^{{\prime} }$-min-mers and an edge exists between two nodes *x* and *y* if the suffix of *x* of size ${k}^{{\prime} }-1$ (that is, its ${k}^{{\prime} }-1$ first minimizers) is equal to the prefix of *y* of size ${k}^{{\prime} }-1$ (that is, its ${k}^{{\prime} }-1$ last minimizers). We defer details about reverse complementation to the ‘Assembler implementation details’ section.

#### Unitig

A unitig (or simple path) is a maximal-length sequence of distinct nodes in the graph such that, given a unitig length *n*, for all nodes except the first and the last one, the in- and out-degrees of each node are equal to 1, and if *n* > 1, then the out-degree of the first node is 1 and the in-degree of the last node is 1. Singleton nodes (*n* = 1) are also considered to be unitigs.

#### Unitig abundance

We define the unitig abundance as the median abundance of its constituent ${k}^{{\prime} }$-min-mers.

#### mContig

Contigs have the same definition as unitigs, except that they are unitigs obtained after graph simplification. Contigs are first extracted as ordered lists of ${k}^{{\prime} }$-min-mers (a path in the graph). The mContig is constructed by concatenating the first ${k}^{{\prime} }-1$ minimizers of its first ${k}^{{\prime} }$-min-mer and the last minimizer of each following ${k}^{{\prime} }$-min-mer (that is, the sequence of ${k}^{{\prime} }$-min-mers without their ${k}^{{\prime} }-1$ overlapping region). The mContig representation will be used to extract (${k}^{{\prime} }+1$)-min-mers in the multi-${k}^{{\prime} }$ algorithm.

#### Contig

At the end of the assembly process, the mContigs are converted to base-space by concatenating the base-space sequence spanned by the minimizers (see ‘Converting to base-space and assembly post-processing’ for more details).

### Algorithmic components

The overall assembly workflow is given in Fig. 1. Input reads are first converted into their minimizer-space representation (mReads). We then initiate a multi-${k}^{{\prime} }$ assembly algorithm in minimizer space. The following operations are performed during each iteration. The abundance of ${k}^{{\prime} }$-min-mers is determined, and low-abundance ${k}^{{\prime} }$-min-mers, deemed as erroneous, are discarded. An MDBG graph is then constructed, and classical assembly graph simplification steps such as tip clipping and bubble popping are performed. Then an algorithm, termed ‘local progressive abundance filter’, is applied to remove potential inter-genomic repeats, strain variability and complex error patterns. The resulting mContigs are added to the set of mReads for the next iteration. At the end of the multi-${k}^{{\prime} }$ process, reads are mapped to the final mContigs in order to output their polished sequences in base-space. In the following sections, we describe in more detail each of the major steps.

### Multi-*k*′ MDBG assembly

In classical DBG metagenome assembly, the choice of the *k*-mer size is critical. Smaller *k*-mers increase sensitivity, as they recover overlaps between reads from rare species and are less sensitive to sequencing errors. By contrast, larger *k*-mers yield higher-contiguity assemblies by resolving longer repeats as well as avoiding spurious overlaps between close strains. In order to retain the best of both worlds, multi-*k* strategies have been introduced^28^. The assembler typically iterates over *k* values from values *k*_min_ to *k*_max_ by fixed increments. In each iteration, a DBG is constructed from the input reads and the contigs are generated from the previous iteration.

In minimizer space, there are three ways to increase the base-equivalent length of a ${k}^{{\prime} }$-min-mer: decrease the density *d*, increase the minimizer length or increase the value of ${k}^{{\prime} }$. We rule out increasing the minimizer length *k* under the hypothesis that doing so would increase sensitivity to sequencing errors. Changing the density is, in principle, interesting because it affects only the distance between consecutive minimizers; however, this would require the recomputation of all minimizers within the mReads and mContigs for each iteration of the assembler, which would be computationally costly. Therefore, we decided to increase only the ${k}^{{\prime} }$ parameter (the length of the ${k}^{{\prime} }$-min-mer), as this does not require minimizers to be recomputed.

In metaMDBG, we iterate over ${k}^{{\prime} }$ from values ${k}_{\rm{min}}^{{\prime} }$ to ${k}_{\rm{max}}^{{\prime} }$ by increments of 1 (see ‘Choice of parameters’ for the values and a discussion of $({k}_{\rm{min}}^{{\prime} },{k}_{\rm{max}}^{{\prime} })$). The input reads are parsed only once to generate mReads, using fixed minimizer *k* and *d* parameters. Each iteration then extracts ${k}^{{\prime} }$-min-mers from the mReads. Another advantage of this approach is that the base-space sequence of the contigs never needs to be constructed during the intermediate iterations; only the union of mContigs and mReads is used to construct the next graph.

### Estimating *k*′-min-mer abundance and filtering errors

We aim to refine the abundance of each ${k}^{{\prime} }$-min-mer, that is the number of times a ${k}^{{\prime} }$-min-mer is seen in the input reads. Generally, abundance information is used in DBG assemblers to detect and filter out erroneous *k*-mers before graph construction to reduce its complexity and memory consumption. Here, the same philosophy is adapted and further elaborated for ${k}^{{\prime} }$-min-mers. Refined abundances are estimated in two steps. First, before the first graph construction, ${k}^{{\prime} }$-min-mer abundances are collected from raw ${k}^{{\prime} }$-min-mer counts in mReads. Then, at each ${k}^{{\prime} }$ iteration after graph construction, long mContigs, which are unlikely to be erroneous, are examined to refine the abundances of ${k}^{{\prime} }$-min-mers and better detect erroneous ${k}^{{\prime} }$-min-mers. Refined abundances are then propagated to the ${k}^{{\prime} }$-min-mers of the next multi-${k}^{{\prime} }$ iteration.

#### Initial *k*′-min-mer counting and filtering

Even though the MDBG is a lightweight data structure, inserting all erroneous ${k}^{{\prime} }$-min-mers would dramatically increase graph memory consumption and complexity, making its traversal computationally challenging. Therefore, before constructing the graph for the first value of ${k}^{{\prime} }$, we apply an abundance-based filter on ${k}^{{\prime} }$-min-mers to remove the majority of erroneous values. In metagenomics, detecting erroneous ${k}^{{\prime} }$-min-mers is non-trivial, as low-frequency ${k}^{{\prime} }$-min-mers could correspond either to real genomic sequences coming from rare species or to errors. Our idea in this first step is to consider the ${k}^{{\prime} }$-min-mers in the context of the read from which they have been extracted: an estimate of a long read ‘abundance’ is determined and those ${k}^{{\prime} }$-min-mers with very low ‘local’ abundance are filtered out.

More precisely, we first perform ${k}^{{\prime} }$-min-mer counting, which is similar to classical *k*-mer counting in that the number of occurrences of each distinct ${k}^{{\prime} }$-min-mer is determined. Then each read is processed sequentially. We define the read coverage (*R*_cov_) as the median of abundances of all its constituent ${k}^{{\prime} }$-min-mers. We then determine a minimum abundance cutoff *R*_min_ = *R*_cov_ × *β* (where *β* = 0.1, empirically determined). A ${k}^{{\prime} }$-min-mer is discarded if the following two criteria are satisfied: its abundance = 1 and its value is lower than *R*_min_. This removes ${k}^{{\prime} }$-min-mers that are seen only once, which represents the vast majority of erroneous ${k}^{{\prime} }$-min-mers, but only within reads in which *R*_cov_ is greater than 1 / *β*. It is a conservative filter to prevent high memory usage on deeply sequenced data sets. Other potentially erroneous ${k}^{{\prime} }$-min-mers will be detected during the contig-generation process by the ‘local progressive abundance filter’ method described in the next subsection.

#### Refining *k*′-min-mer abundances

After mContigs have been generated (see next section), ${k}^{{\prime} }$-min-mer abundances are refined. We introduce two techniques: abundance smoothing and long contig ${k}^{{\prime} }$-min-mer rescuing. The smoothing step is performed first. The abundance of an mContig *C*_cov_ is computed as the median abundance of its constituent ${k}^{{\prime} }$-min-mers. In the mContig, the abundance of each ${k}^{{\prime} }$-min-mer is then set to the refined abundance *C*_cov_. Long mContigs (having >2${k}^{{\prime} }$ ${k}^{{\prime} }$-min-mers) are unlikely to contain any erroneous ${k}^{{\prime} }$-min-mers. If a ${k}^{{\prime} }$-min-mer with an abundance of 1 is present in a long mContig, it is rescued by incrementing its refined abundance by 1 so that it will pass the pre-filtering performed in the next iteration.

#### Propagating refined abundance to the next *k*′ iteration and filtering

At the beginning of each subsequent multi-${k}^{{\prime} }$ iteration except the initial one (${k}^{{\prime} } > {k}_{\rm{min}}^{{\prime} }$), we estimate ${k}^{{\prime} }$-min-mers based on the refined abundance of (${k}^{{\prime} }$ − 1)-min-mers determined in the previous iteration. A ${k}^{{\prime} }$-min-mer contains two overlapping (${k}^{{\prime} }$ − 1)-min-mers for which the refined abundance is known. We define the refined abundance of a ${k}^{{\prime} }$-min-mer as the minimum of its two (${k}^{{\prime} }$ − 1)-min-mer abundances. We use the minimum instead of the average because if one of the two (${k}^{{\prime} }$ − 1)-min-mers is erroneous, we do not wish its abundance to be raised by the other potentially correct one. This refined abundance propagation technique has several advantages. Firstly, it improves ${k}^{{\prime} }$-min-mer abundance estimation over using abundances determined from reads alone. Secondly, it prevents ${k}^{{\prime} }$-min-mer abundances from collapsing to one (or even zero) when determined from reads alone as we increase ${k}^{{\prime} }$; indeed, long ${k}^{{\prime} }$-min-mers tend to be underrepresented because they are more likely to contain a sequencing error or to be longer than the mReads themselves. Finally, refined abundances allow us to assign an abundance estimate to ${k}^{{\prime} }$-min-mers that exist only in mContigs and not in mReads.

After the ${k}^{{\prime} }$-min-mer refined abundances have been determined, all ${k}^{{\prime} }$-min-mers with a single occurrence are discarded. As we progress in the multi-${k}^{{\prime} }$ process, we notice that erroneous ${k}^{{\prime} }$-min-mers tend to occur only once, whereas correct ${k}^{{\prime} }$-min-mers tend to be rescued and refined to abundances of two or more.

### Local progressive abundance filtering

In this section, we introduce a key component of our contig-generation process that performs progressive abundance filtering to simplify parts of the assembly graph corresponding to abundant organisms (typically above 10–20x coverage). We first explain the rationale and then present the algorithmic details.

We generate contigs by examining the abundances of organisms in the assembly graph through the abundances of unitigs. Recall that a unitig is a maximal-length, non-branching path in the assembly graph. Nearly all unitigs of abundant organisms cluster together into a single large connected component of the assembly graph owing to inter-genomic repeats and chimeric reads in HiFi samples. These two effects increase the complexity of the graph and make assembly challenging. By performing graph simplifications using abundance information, we are able to sidestep both issues.

In principle, some abundant organisms could be separated in silico from the large component of the assembly graph by using an abundance filter; for instance, by removing all nodes with an abundance lower than half that of the organism’s abundance. This is because most of the erroneous overlaps have low coverage: chimeric reads are rare and most inter-genomic repeats are spanned by rare species, so removing the corresponding low-abundance graph nodes will remove those repeats. Filtering using a local abundance criteria has additional advantages: it can remove large stretches of sequencing errors as well as strain variability. However, designing such a filter is not straightforward.

In complex areas of an assembly graph, unitigs tend to be fragmented and their abundances may be under-estimated, resulting in correct unitigs being filtered out whenever removal is based on length or, more critically, absolute abundance. The abundances of chimeric or rare species unitigs in complex areas also tend to be under-estimated^28^. Our solution is to filter out unitigs by iterating over abundance cutoffs, from low to high. At some point in the iterative process, fragmented but correct unitigs will be linked to longer ones and thus successfully rescued.

An unpractical but simple algorithm that illustrates our contig-generation process is as follows. Sort the MDBG unitigs *u*_1_, …, *u*_*n*_ from the most abundant (*u*_1_) to the least abundant (*u*_*n*_). Iterate the following procedure from *i* = 1…*n*. Consider the abundance, *a*_*i*_, of *u*_*i*_ and fix a local abundance cutoff *U*_*i*,cut_ = *a*_*i*_ × *β* (with *β* values in the range of 0.1–0.5; in the real algorithm we will set it to 0.5). Create a copy, ${G}^{{\prime} }$, of the MDBG. For *t* = 1 to *t* = *U*_*i*,cut_, repeat the procedure of removing all unitigs with an abundance less than *t* from ${G}^{{\prime} }$ and then re-compact ${G}^{{\prime} }$. Finally, at *t* = *U*_*i*,cut_, collect the unitig ${u}^{{\prime} }$ in ${G}^{{\prime} }$ that contains *u*. If ${u}^{{\prime} }$ does not contain any ${k}^{{\prime} }$-min-mer from a previously returned contig, then return it as a contig.

Performing assembly with the above procedure for every unitig would be costly and redundant. Instead, in this work, a progressive abundance filter is applied once to the whole graph from thresholds *t* = 1 to *t* = *t*_max_ (see ‘Progressive abundance filtering’) instead of per unitig. At each step, we collect the set of unitigs from the graph. This results in multiple sets of unitigs (${S}_{1},\ldots,{S}_{{t}_{\rm{max}}}$), each corresponding to a single threshold, *t*. A subsequent algorithm iterates over the sets (*S*_*t*_) and non-redundantly outputs all unitigs that are above a well-chosen abundance threshold at each step (see ‘Generating mContigs’).

#### Progressive abundance filtering

This process (Algorithm 1) iterates over abundance thresholds, simplifying and compacting the graph and then removing unitigs that are below the current threshold, saving the remaining unitigs.

Specifically, the algorithm iterates from abundance threshold *t* = 1 to *t* = *t*_max_ (line 3), where *t*_max_ is the abundance of the most abundant unitig in the initial graph. The graph is simplified (line 4, see ‘Graph simplification’ below for details). The graph is then compacted (line 5) and unitigs are collected into a set, *S*_*t*_ (line 6). Finally, unitigs with an abundance ≤ *t* are discarded (line 7) from the graph and we move to the next iteration of *t*.

#### Graph simplification

The simplification step includes two processes: tip clipping and superbubble popping. Tips of 50 kbp or smaller are disconnected from the graph. We do not remove them here as they may either be erroneous or belong to a rare species. These tips are removed at the end of the assembly process if they have a high identity with another contig. Superbubbles of length 50 kbp or smaller are detected in *O*(∣*Edges*∣ + ∣*Nodes*∣) average time following a previously defined algorithm^29^, and the path with maximum abundance is retained.

**Algorithm 1** Progressive abundance filtering.

**Input:** MDBG G

**Output:** ${S}_{1},\ldots,{S}_{{t}_{\rm{max}}}$ sets of unitigs along with their abundance information

1: *S* ← {}

2: *t* ← 1

3: **while** *t* ≤ *t*_max_ **do**

4:  *G* ← *Simplify*(*G*) ⊳ Tip clipping, bubble popping

5:  *G* ← *Compact*(*G*) ⊳ Compact the graph and calculate median ${k}^{{\prime} }$-min-mer abundance of unitigs

6:  *S*_*t*_ ← *Unitigs*(*G*)

7:  Remove unitigs with abundance ≤ *t* from *G*

8:  *t* ← *t* + 1

9: **end** **while**

10: return *S*

#### Generating mContigs

This process iterates over all sets of unitigs (*S*_*t*_) starting from the one with the highest abundance cutoff, ${S}_{{t}_{\rm{max}}}$. For each set, unitigs and their abundances are scanned in no particular order and a unitig, *u*, is returned if its abundance, *a*, is greater than some threshold. We call mContigs the set of returned unitigs (in line with typical genome assembly usage, where a contig is generally a unitig within the simplified assembly graph). The complete process is described in Algorithm 2.

Specifically, at each iteration, a unitig, *u*, from *S*_*t*_ along with its abundance, *a*, is added to the final set of mContigs if it does not share any ${k}^{{\prime} }$-min-mer with any other unitig already in mContigs and also if its abundance, *a*, is greater than *a* × *t* / *β* (line 6). The ${k}^{{\prime} }$-min-mers within *u* are recorded in a set of outputted nodes to prevent redundancy (lines 7 and 8).

Here, the sets of unitigs (*S*_*t*_) are iterated from the large abundance threshold to the low-abundance threshold rather than the opposite. This is done to ensure that we always output unitigs in their longest possible form. To illustrate, consider what would happen if we had started with the lowest threshold. There would be no way of knowing whether a given unitig has been maximally merged with some other unitig(s) after our abundance-filtering and graph-simplifications steps. For example, at the abundance threshold of three, all unitigs with an abundance of six would be output because they pass the local abundance threshold of 3 / 0.5 = 6. However, among them, there may also be fragmented unitigs that belong to a more abundant species (for instance, of abundance ten) that are ‘waiting’ to be merged with other unitigs after more substantial simplifications (for instance, at *t* = 4 or *t*  = 5). Iterating from the large threshold to the low threshold solves this issue.

**Algorithm 2** Generating mContigs.

**Input:** ${S}_{1},\ldots,{S}_{{t}_{\rm{max}}}$ sets of unitigs

**Output:** mContigs

1: *t* ← *t*_max_

2: *C* ← {} ⊳*C* is the set of ${k}^{{\prime} }$-min-mers in the mContigs

3: *β* ← 0.5

4: **while** *t* ≥ 1 **do**

5:  **for** each unitig *u* (with abundance *a*) in *S*_*t*_ **do**

6:   **if** $C\cap nodes(u)={{\emptyset}}$ and *a* > *t* / *β* **then**

7:    Output *u*

8:    * C* ← *C* ∪ *nodes*(*u*)

9:   **end** **if**

10:  **end** **for**

11:  *t* ← *t* − 1

12: **end** **while**

### Converting to base-space and assembly post-processing

At the end of the multi-${k}^{{\prime} }$ process, the base-space representation of mContigs (that is, the actual nucleotide sequences and not their minimizer-space representation) is constructed by gathering the base sequences corresponding to all mContigs ${k}^{{\prime} }$-min-mers from the original reads. This is followed by two post-processing steps. A contig polishing step fixes sequencing errors in contigs (mostly homopolymers), and an optional duplication-purging step removes similar contigs corresponding to close strains.

#### Constructing contig base sequences

This step converts mContigs (that is, the minimizer-space representation of contigs) to actual nucleotide-space contigs. The idea is to choose a particular ${k}^{{\prime} }$ value, collect ${k}^{{\prime} }$-min-mer nucleotide sequences from the original reads and then reconstruct contig nucleotide sequences by aggregating the ${k}^{{\prime} }$-min-mer nucleotide sequences. This is a generalization of the method presented in a previous work^18^ to the multi-${k}^{{\prime} }$ setting, made more accurate by using read mapping. Indeed, a ${k}^{{\prime} }$-min-mer can be generated by multiple different nucleotide sequences. Hence, collecting the ‘wrong’ nucleotide sequence could yield errors in contigs. Large values of ${k}^{{\prime} }$ yield more specific ${k}^{{\prime} }$-min-mers, minimizing such errors. However, some of these long ${k}^{{\prime} }$-min-mers may exist only in mContigs and not in mReads; therefore, their nucleotide sequences cannot be constructed with certainty. We use ${k}^{{\prime} }={k}_{\rm{min}}^{{\prime} }$ to ensure that all contig ${k}^{{\prime} }$-min-mers are indeed present in the reads. To collect the ‘true’ nucleotide sequence of each contig ${k}^{{\prime} }$-min-mer, mReads are first mapped to mContigs. The ${k}^{{\prime} }$-min-mer sequences are then collected from the reads that best match the contigs. The read mapping strategy in minimizer space is described as follows.

The mContigs are firstly indexed to create a set of ${k}^{{\prime} }$-min-mer seeds: each mContig ${k}^{{\prime} }$-min-mer is stored as a key in a hash table with the associated values being a list of contig positions, represented as pairs {*c*_*i*_, *c*_*p*_}, where *c*_*i*_ is the contig identifier and *c*_*p*_ is the ${k}^{{\prime} }$-min-mer position in *c*_*i*_. Then, mReads are scanned, and for each mRead ${k}^{{\prime} }$-min-mer found in one or more mContigs, its mContig position(s) are retrieved as seeds for potential mappings. The seeds are extended maximally: we iterate over the mRead ${k}^{{\prime} }$-min-mers (to the left and to the right of the seed) and extend mappings as long as subsequent ${k}^{{\prime} }$-min-mers continue to be the same as those that follow in the mContig(s). The result is a set of intervals (made non-redundant) indicating maximal matches between the current mRead and one or several mContigs. Then another hash table with contig ${k}^{{\prime} }$-min-mer positions {*c*_*i*_, *c*_*p*_} as keys (here *c*_*p*_ is the position of the seed in the mContig, one position per mapping obtained) maintains the maximal matches as triplets {*r*_*i*_, *r*_*p*_, *m*}, where *r*_*i*_ is the read identifier, *r*_*p*_ is the position of the seed ${k}^{{\prime} }$-min-mer in *r*_*i*_ and *m* is the length of the longest match.

The overall mapping algorithm is thus quadratic over the number of ${k}^{{\prime} }$-min-mers in each mRead. However, in practice, this number is close to 45, making the algorithm highly practical. We process mReads twice, in forward and reverse order, to handle reverse complements. The output of the algorithm is exactly one read ${k}^{{\prime} }$-min-mer position for each contig ${k}^{{\prime} }$-min-mer position.

The reads are then parsed in nucleotide space and their ${k}^{{\prime} }$-min-mers are extracted. If a ${k}^{{\prime} }$-min-mer is reported as a best match during the above mapping procedure, then we collect the substring of the read corresponding to that ${k}^{{\prime} }$-min-mer. To deal with overlaps between successive ${k}^{{\prime} }$-min-mers in mContigs, we also record the position of the second and second-to-last minimizers within each ${k}^{{\prime} }$-min-mer. We finally parse mContigs and concatenate the sequences associated with their ${k}^{{\prime} }$-min-mers, making sure to discard overlaps.

#### Contig polishing

We perform an additional polishing step on the base-level representation of contigs to remove sequencing errors. We re-implemented a strategy akin to racon^19^: reads are first uniquely assigned to contigs using minimap2, contigs are then split into non-overlapping windows of 500 nucleotides and fragments of reads that map to each window are collected. Finally, a consensus sequence for each window is created by partial order alignment using the SPOA library^19^.

Our polishing differs from that of racon, in particular in the following two aspects. The first is how we select reads in the case of multiple mappings. We noticed that longer alignments are not necessarily the best ones, but that alignment identity must also be considered. We score alignments using the metric *MS* = *alignLength* *×* *alignIdentity* and for each read, retain only the alignment that maximizes *MS*. The second is a reduction of memory usage. We limit the number of read fragments used to correct a window. With accurate long reads, we noticed that using only 20 fragments is sufficient to produce a high-quality consensus. We also reduce the memory required to store the read fragments by partitioning the contigs and the reads that map onto them on the disk, processing one partition at a time. The memory required to store the read fragments of a contig is estimated by multiplying the contig length by the contig coverage (estimated from the initial read mapping). Contigs are processed sequentially and written into a partition file until the memory required to process the partition exceeds 6 GB. The current partition is then closed and a new one is started. A structure in memory records the association of contigs to partitions. Similarly, reads are then processed and written to the partition of their best-matching contig. This results in an approximately 100-fold reduction in memory usage compared to the original racon implementation for the sheep rumen data set.

#### Strain duplication purging

Sequence duplications in contigs caused by strain variability are detected by all-versus-all contig mapping using wfmash^30^. Contigs longer than 1 Mbp are left untouched and are used as templates to remove duplications that are present in shorter contigs. For those shorter contigs, we remove any part overlapping with a ≥1 Mbp contig when the overlap nucleotide alignment identity is greater than 99%.

### Choice of parameters

Our method has four critical parameters: the minimizer size, the minimizer density and the starting and ending ${k}^{{\prime} }$-min-mer size, ${k}_{\rm{min}}^{{\prime} }$ and ${k}_{\rm{max}}^{{\prime} }$.

The minimizer size and density were both set empirically to 13 and 0.005, respectively (that is, roughly 0.5% of total *k*-mers are used as minimizers). In our tests, using such short minimizers leads to better results than using longer minimizers, possibly because they are less sensitive to sequencing errors.

The starting $${k}^{{\prime} }$$-min-mer size, *k*′_min_ was fixed to 4. Using $${k}^{{\prime} }$$ values less than 4 creates assembly graphs that have high complexity, resulting in highly fragmented contigs. The ending $${k}^{{\prime} }$$-min-mer size, $${k}_{\rm{max}}^{{\prime} }$$, is a function of the sample median read length: $${k}_{\rm{max}}^{{\prime} }=$$$$medianReadLength\,\times \,density\,\times \,2$$.

With density 0.005 and *k*′_min_ = 4, the assembler initially considers overlaps between reads with lengths of $\frac{4-1}{0.005}=600$ bases on average. It then iteratively increases the overlap length, in increments of 200 bases, until finally processing overlaps of twice the median length of the reads.

### Anaerobic digester sample extraction and long-read DNA sequencing

Facility operators obtained three biomass samples directly from an anaerobic digester reactor that was digesting food waste at weeks 1, 20 and 40 of a year-long sampling campaign. The samples were shipped in ice-cooled containers to the University of Warwick. Upon receipt, they were stored at 4°C, subsampled into several 1–5 ml aliquots within a few days and then stored in 1.8 ml cryovials at −80°C. Samples were defrosted at 4°C overnight before DNA extraction. DNA was extracted from a starting mass of 250 mg of anaerobic digester sludge using the MP Biomedical FastDNA SPIN Kit for Soil (cat no. 116560200) and a modified manufacturer’s protocol.

DNA size was assessed using a FemtoPulse (Agilent). The Pacific Biosciences protocol ‘Preparing 10 kb Library Using SMRTbell Express Template Prep Kit 2.0 for Metagenomics Shotgun Sequencing’ was used to create libraries from 1.5 µg of DNA. In most cases, the DNA was already 10 kb or smaller. Sample AD2W40 was slightly larger; therefore, the DNA was sheared using a g-TUBE (Covaris) for one library and unsheared for a second library. Libraries were not pooled because of the large number of reads that were desired. Sequencing was performed using a Sequel II sequencer (Pacific Biosciences) using version 8M SMRT cells and version 2.0 sequencing reagents with 30 h movies and a 2 h pre-extension time to generate circular consensus sequencing reads.

### Assembling data sets, mapping reads and binning contigs

We ran all assemblers with 16 central processing unit threads. We used the default parameters of metaMDBG for all assemblies (minimizer size, 13; density, 0.005). We ran hifiasm-meta with the default parameters on real data and with the option ‘–force-preovec’ on the mock communities as suggested by the authors. We only used the hifiasm-meta primary assembly of polished contigs (p_ctg.gfa), as adding alternate contigs reduced the overall MAG quality. We ran metaFlye with the options ‘–meta’ and ‘–pacbio-hifi’ for HiFi data sets and with the option ‘–nano-hq’ for Nanopore data sets. We used the command ‘/usr/bin/time -v’ to obtain wall-clock runtime and peak memory usage. All tools that were used and the complete command line instructions are available in Supplementary Table S2.

To determine the fraction of reads that were mapped to the assemblies, we used ‘minimap2 -x asm20’ as suggested in the metaFlye study^10^. We filtered out reads in which all of the alignments were shorter than 80% of its length, and we assigned each remaining read to a unique contig through its longest alignment (breaking ties arbitrarily). To estimate contig coverage across samples before binning, we used the command ‘minimap2 -ak19 -w10 -I10G -g5k -r2k –lj-min-ratio 0.5 -A2 -B5 -O5,56 -E4,1 -z400,50 ∣ samtools sort -o outut.bam’ as proposed in the hifiasm-meta article^14,31^. We input the resulting binary alignment map to the program jgi_summa_rsize_bam_contig_depths of MetaBAT2 to obtain contig coverage profiles across samples.

We performed contig binning using MetaBAT2 (ref. ^32^), with default parameters and a fixed seed (–seed 42) for reproducibility. As MetaBAT2 may bin strains from the same species, creating a single apparently contaminated MAG, we separated all circular contigs of 1 Mb or longer before binning the remaining contigs, as suggested in the hifiasm-meta study^14^.

### Quality assessment of assemblies

We used CheckM (v.1.1.3) to assess the quality of all MAGs and circular contigs longer than 1 Mbp. We used viralVerify^21^ (v.1.1) to identify plasmids and viruses in each assembly. We considered only contigs shorter than 500 kbp with prediction scores higher than five. Annotations labeled as ‘Plasmid’ or ‘Uncertain - plasmid or chromosomal’ were considered as plasmids and, similarly, annotations labeled as ‘Virus’ or ‘Uncertain - viral or bacterial’ were considered as viruses. We used checkV^22^ to assess the quality of viral contigs. We used Barrnap (https://github.com/tseemann/barrnap), and Infernal^33^ to predict, respectively, rRNA and tRNA genes from circular contigs. We filtered out annotations with E-values over 0.01. A total of 437 (96%) near-complete circular contigs found by metaMDBG had one copy of the 5S, 16S and 23S genes and at least 18 tRNA genes, compared to 96.6% for hifiasm-meta and 98.5% for metaFlye (Supplementary Table S12).

### Assessment of completeness and fragmentation of assemblies with reference sequences

We used the following process to assess the completeness and fragmentation of assemblies when reference genomes are available (mock reference genomes or near-complete circular contigs). We used wfmash to align contigs against the reference sequences. Alignments with less than 99% identity were filtered out. Alignments were ordered by their matching score from *MS* = *alignLength* × *alignIdentity* (best score first). We considered alignment identity to improve contig assignment to similar strains. Alignments were then processed sequentially and contigs were uniquely assigned to references. During this process, we check whether a reference is complete or not, meaning that at least 99% of its positions are covered by contigs. We prevent other contigs from being assigned to a complete reference. Moreover, we prevent a contig from being assigned to a reference if more than 30% of its matching positions are already covered by another contig. In this case, we first try to assign this contig to another reference. References with less than 70% completeness were considered to be missed by the assembler.

### Taxonomic classification of MAGs recovered from anaerobic digester samples

The phylogenetic tree in Fig. 2 was built using fasttree^34^ from the output alignment of GTDB-Tk v.2.1.0 (ref. ^35^) on near-complete-quality MAGs of all three assemblers for the anaerobic digester data set. Concurrent diversity coverage between the different assemblers was explored at different taxonomic levels from genus to domain. To do so, it is necessary to first address MAGs for which no annotation is available at a given taxonomic rank. A pair of unannotated MAGs may or may not share the same taxa. A first pass based on tree topology allows us to select neighboring MAGs as candidates for sharing the same unknown taxa. As a second step, we compute the relative evolutionary distance (RED) using the R library Castor v.1.7.3 (ref. ^36^). Following guidelines from GTDB, we use their median RED values for each taxon in order to decide whether to group unknown MAGs together. We then find the best ancestor for each unknown MAG in terms of its RED being nearest to the corresponding taxon’s median RED. If they share the same best ancestor, then we group them together; otherwise, we split them into distinct unknown taxa. Tree manipulation and representation are carried out using the libraries ggtree v.2.4.1 (ref. ^37^), treeio v.1.14.3 (ref. ^38^) and ggtreeExtra version 1.0.2 (ref. ^39^).

#### Assembler implementation details

During transformation to minimizer space, reads are homopolymer-compressed^40^. We handle reverse recomplements in a manner that is similar but slightly different than classical DBG assembly. We consider canonical ${k}^{{\prime} }$-min-mers by comparing to its reverse (not its reverse complement). The first minimizer of each is compared; the ${k}^{{\prime} }$-min-mer with the smallest minimizer is selected as the canonical representative. In the case of equality, the second minimizer of each is compared, and so on. Note that minimizers are also considered in their canonical representations, which, in this case, is identical to the classical technique. A minimizer is in canonical form if its forward sequence is lexicographically equal to or smaller than its reverse-complement sequence.

### Reporting summary

Further information on research design is available in the Nature Portfolio Reporting Summary linked to this article.

## Online content

Any methods, additional references, Nature Portfolio reporting summaries, source data, extended data, supplementary information, acknowledgements, peer review information; details of author contributions and competing interests; and statements of data and code availability are available at 10.1038/s41587-023-01983-6.

## Supplementary information

Reporting Summary

Supplementary Tables Supplementary Tables 1–12

## Data Availability

All data sets used in this study were downloaded from the NCBI Sequence Read Archive; accession numbers are given in Supplementary Table S1. Zymo-HiFi mock reference genomes are available at https://s3.amazonaws.com/zymo-files/BioPool/D6331.refseq.zip. ATCC mock reference genomes are available at https://www.atcc.org/products/msa-1003.

## Code availability

MetaMDBG is available at https://github.com/GaetanBenoitDev/metaMDBG. The analysis scripts used in this study to compare assemblers are available at 10.5281/zenodo.8321179.
