# Supplementary material for: High-quality metagenome assembly from long accurate reads with metaMDBG
Source: Nat Biotechnol. 2024 Jan 2;42(9):1378–83. doi: 10.1038/s41587-023-01983-6 (PMC11392814; doi:10.1038/s41587-023-01983-6)
Supplement: Supplementary file 1 — Reporting Summary [file 41587_2023_1983_MOESM1_ESM.pdf]

nature portfolio

Corresponding author(s): Dr Christopher Quince

Last updated by author(s): Sep 6, 2023

Reporting Summary

Nature Portfolio wishes to improve the reproducibility of the work that we publish. This form provides structure for consistency and transparency in reporting. For further information on Nature Portfolio policies, see our Editorial Policies and the Editorial Policy Checklist.

Statistics

For all statistical analyses, confirm that the following items are present in the figure legend, table legend, main text, or Methods section.

n/a	Confirmed	
☐	☒	The exact sample size (*n*) for each experimental group/condition, given as a discrete number and unit of measurement
☒	☐	A statement on whether measurements were taken from distinct samples or whether the same sample was measured repeatedly
☒	☐	The statistical test(s) used AND whether they are one- or two-sided<br>*Only common tests should be described solely by name; describe more complex techniques in the Methods section.*
☐	☒	A description of all covariates tested
☒	☐	A description of any assumptions or corrections, such as tests of normality and adjustment for multiple comparisons
☐	☒	A full description of the statistical parameters including central tendency (e.g. means) or other basic estimates (e.g. regression coefficient) AND variation (e.g. standard deviation) or associated estimates of uncertainty (e.g. confidence intervals)
☐	☒	For null hypothesis testing, the test statistic (e.g. *F*, *t*, *r*) with confidence intervals, effect sizes, degrees of freedom and *P* value noted<br>*Give P values as exact values whenever suitable.*
☒	☐	For Bayesian analysis, information on the choice of priors and Markov chain Monte Carlo settings
☒	☐	For hierarchical and complex designs, identification of the appropriate level for tests and full reporting of outcomes
☒	☐	Estimates of effect sizes (e.g. Cohen's *d*, Pearson's *r*), indicating how they were calculated

Our web collection on statistics for biologists contains articles on many of the points above.

Software and code

Policy information about availability of computer code

Data collection	Software not used for data collection
Data analysis	The following open source programs were used in the analysis:<br>metaMDBG v0.3<br>hifiasm-meta v0.2-r058<br>metaflye v2.9-b1768<br>minimap2 v2.21-r1071<br>samtools v1.16.1<br>jgi_summarize_bam_contig_depths v2<br>metabat2 v2<br>CheckM v1.2.1<br>CheckV v1.0.1<br>dRep v3.2.2<br>Infernal v1.1.4<br>Barrnap v0.9<br>Viralverify v1.1<br>wfmash v0.10.0<br>R package fasttree v2.16<br>R package gtdbtk v2.1.0<br>R package Castor v1.7.3<br>R package ggtree v2.4.1

R package treeio v1.14.3
R package ggtreeExtra v1.0.2

Custom code was submitted to repositories: https://github.com/GaetanBenoitDev/metaMDBG and https://github.com/GaetanBenoitDev/MetaMDBG_Manuscript.

For manuscripts utilizing custom algorithms or software that are central to the research but not yet described in published literature, software must be made available to editors and reviewers. We strongly encourage code deposition in a community repository (e.g. GitHub). See the Nature Portfolio guidelines for submitting code & software for further information.

Data

Policy information about availability of data

All manuscripts must include a data availability statement. This statement should provide the following information, where applicable:

- Accession codes, unique identifiers, or web links for publicly available datasets
- A description of any restrictions on data availability
- For clinical datasets or third party data, please ensure that the statement adheres to our policy

All datasets used is this study were downloaded from NCBI Sequence Read Archive (SRA) with accession numbers given in Table 1. Zymo mock reference genomes are available at https://s3.amazonaws.com/zymo-files/BioPool/D6331.refseq.zip. ATCC mock reference genomes are available at https://www.atcc.org/products/msa-1003.

Human research participants

Policy information about studies involving human research participants and Sex and Gender in Research.

Reporting on sex and gender	Not relevant
Population characteristics	Not relevant
Recruitment	Not relevant
Ethics oversight	Not relevant

Note that full information on the approval of the study protocol must also be provided in the manuscript.

Field-specific reporting

Please select the one below that is the best fit for your research. If you are not sure, read the appropriate sections before making your selection.

☒ Life sciences ☐ Behavioural & social sciences ☐ Ecological, evolutionary & environmental sciences

For a reference copy of the document with all sections, see nature.com/documents/nr-reporting-summary-flat.pdf

Life sciences study design

All studies must disclose on these points even when the disclosure is negative.

Sample size	Sample sizes in terms of biological samples were restricted by available data sets that were publicly available and relevant at time of submission. That was two mocks, four from human gut, three AD and one sheep rumen. However, these sample sizes are irrelevant as we do not draw conclusions between sample types. Instead our statistics are based on reconstructed genomes within a sample under different conditions which is an intrinsic property of the sample rather than something we can control.
Data exclusions	No data was excluded.
Replication	Replication in the sense that three independent sequencing studies were analysed (AD, humam gut and sheep rumen).
Randomization	We did not have participants assigned to treatment groups so that randomization is not relevant.
Blinding	Similarly blinding cannot be performed in the absence of treatment groups or subjects.

Reporting for specific materials, systems and methods

We require information from authors about some types of materials, experimental systems and methods used in many studies. Here, indicate whether each material, system or method listed is relevant to your study. If you are not sure if a list item applies to your research, read the appropriate section before selecting a response.

Materials & experimental systems

n/a	Involved in the study
☒	☐ Antibodies
☒	☐ Eukaryotic cell lines
☒	☐ Palaeontology and archaeology
☒	☐ Animals and other organisms
☒	☐ Clinical data
☒	☐ Dual use research of concern

Methods

n/a	Involved in the study
☒	☐ ChIP-seq
☒	☐ Flow cytometry
☒	☐ MRI-based neuroimaging
